# Transition to Adult Care in Children on Long-Term Ventilation

**DOI:** 10.3389/fped.2020.548839

**Published:** 2020-09-30

**Authors:** Alessandro Onofri, Alexander Broomfield, Hui-leng Tan

**Affiliations:** ^1^Pediatric Pulmonology & Respiratory Intermediate Care Unit, Sleep and Long-Term Ventilation Unit, Academic Department of Pediatrics, Bambino Gesü Children's Hospital IRCCS, Rome, Italy; ^2^Willink Biochemical Genetics Unit, Manchester Center for Genomic Medicine, St. Mary's Hospital, Central Manchester Foundation Trust, Manchester, United Kingdom; ^3^Department of Paediatric Respiratory Medicine, Royal Brompton Hospital, London, United Kingdom

**Keywords:** transition, pediatric to adult services, long term ventilation, barriers and facilitators, Home mechanical ventilation

## Abstract

The number of children on long-term ventilation (LTV) has exponentially increased over the past few decades. Improvements in management of ventilation coupled with improvements in standards of medical care are increasingly allowing young people on LTV to survive into adulthood. The process of transition from the pediatric to the adult healthcare system is challenging and requires special attention.

This review aims to provide an overview on transition to adult care for children on LTV. Firstly, examining effective models of transition in other childhood onset chronic conditions as a template, whilst highlighting the unique aspects of transition in LTV patients and secondly, summarizing the main relevant findings in the literature on the topic and emphasizing the importance of a multidisciplinary approach to this process.

## Introduction

Improving medical care has resulted in a progressive increase in the number of children with chronic diseases reaching adulthood and more than 85% of children with chronic illness now will need to transition to adult services. These patients can be broadly stratified according to their level of need: ~5% have complex needs; 25% have complex chronic conditions; whilst the remaining 70% are patients with chronic conditions with good control (1). Children on LTV tend to fall into the first two categories. Since the initial use of LTV in children to treat chronic respiratory failure in the 1980s, its use has expanded and it is now widely used in three main scenarios: neuromuscular disease, upper airway conditions, and abnormalities of central respiratory drive, e.g., congenital central hypoventilation syndrome (2, 3). LTV is effective in improving respiratory parameters and thoraco-abdominal coordination during sleep, reduces the workload of the respiratory system, palliates symptoms, and in certain conditions, has been shown to increase life expectancy (4–7). More widespread provision of LTV support, in conjunction with improved survival means that increasing numbers of patients on LTV will be transitioning to adult services.

This process of transition from pediatric to adult healthcare systems is one of the most challenging periods for patients. Blum et al. (8) defined “transitional care” as “the purposeful, planned movement of adolescents, and young adults with chronic physical and medical conditions from child-centered to adult-oriented healthcare systems”. Adolescence is recognized as a vulnerable period due to the physiological, emotional, and psychosocial changes occurring. Adolescents with chronic diseases not only have to deal with the problems and needs that healthy adolescents have, such as the need to make new friends and form close relationships, school struggles, new intellectual interests, physical, and sexual attractiveness, setting of goals and future objectives; they similarly face choices about risky behaviors (alcohol, tobacco, and drugs) whilst facing the potential for greater adverse health outcomes from these behaviors (9). Therefore, it is essential to consider “transition” from pediatric to adult services in the global context of a period of multifactorial change for the patient.

Most healthcare professionals are trained in either pediatric or adult medicine and often feel less confident when managing adolescents (10). An international cross-jurisdictional policy scoping review by Hepburn et al. (11) examined all publicly available national government documents detailing transition strategies, from nine countries with comparable healthcare systems. They found that transition has received little government attention and only two of the nine countries had an operational strategy regarding transition. Whilst many hospitals have developed transition programs, most are institution and disease specific and not universally accessible (11). They are also often limited in terms of capacity and face funding barriers.

Doing transition well, is arguably even more important for patients with complex diseases, such as those on LTV, however, it is also in these very patients where inadequacy of transitional care is more evident. It is instructive to examine the current differing care models that already exist for the transition process, for different chronic pediatric diseases to see what is applicable to patients on LTV.

## Congenital Heart Disease

Advances in management of congenital heart disease (CHD) have resulted in a significant improvement in survival, particularly in those with more complex conditions (12). Almost all international guidelines on the care of CHD patients now include recommendations regarding transition. The lack of adult specialists and of adequate facilities in adult hospitals for patients with CHD has been highlighted as an issue. Infrastructure and staff requirements for specialist “grown up CHD” centers have been defined (13). Specific formal training in the subspeciality has been identified as being essential for adult and pediatric cardiologists. European guidelines advocate at least one assessment in a specialist center where the CHD specialist can assess the required level of care and frequency of follow-up, stratifying patients to those requiring continued specialist follow up, those who can have shared care with general adult cardiac services, and those who can be managed in general (non-specialist) cardiac clinics, with access to specialist care when required (13). American guidelines highlight the importance of delivering developmentally appropriate transition education to teach patients and families about the expectations and concerns regarding their specific CHD condition, as well as the skills to navigate the adult healthcare system (14). A trial of a transition intervention consisting of a 1 h nurse-led one-on-one teaching session resulted in significant improvement in cardiac knowledge and self-management scores 6 months post-intervention (15).

## Cystic Fibrosis

Considerable investment in clinical and basic science research has transformed cystic fibrosis (CF) from an early lethal condition to a chronic illness. Mathematical modeling of UK CF registry survival data predicts that over half of babies born today, can expect to survive into their fifth decade, if not longer (16). The period of transition to adult care is a crucial time in the natural history of CF patients. Late CF related complications such as infertility, gastro-intestinal cancer, and infections by multi drug resistant pathogens such as atypical Mycobacterium, have emerged. Psychological issues associated with worsening lung function, reduced life expectancy, and diminished fertility may manifest and adolescents may be influenced by peers to try risky behaviors such as smoking which could have a significant detrimental impact on their lung function (17). The response of the CF community to these challenges has been commendable. Strong advocacy and emphasis on research into health care transition have meant that CF services now offer one of the highest quality transition processes amongst all the chronic diseases and there is much one can learn from them (18). North American and European standards of care and consensus guidelines for the care of CF patients include recommendations on transition (19–21). Some features highlighted include:

(a) Starting discussions about transition early.
(b) To aim for patient transfer to occur between the ages of 16–18 years, but this should incorporate a degree of flexibility, reflecting the patient's developmental maturity, and health status.
(c) The need for close cooperation between the pediatric and adult centers, with continuity of care being facilitated by the joint adoption of the same diagnostic and treatment protocols tailored to specific age groups.
(d) A formal transition clinic where there is participation of both pediatric and adult teams.
(e) Referral only by letter is suboptimal.
(f) A comprehensive formalized transfer report including input from every member of the multidisciplinary team must be provided.

## Type 1 Diabetes Mellitus

Unlike CHD and CF, survival into adulthood has not in the main been an issue in Type 1 Diabetes Mellitus (T1DM) meaning its transition processes are some of the most well established. Alassaf et al. (22) found that young persons with complicated health histories and less education are more likely to have poorer diabetic outcomes following transition to adult care. Different models of transition such as having specific young adult clinics, a dedicated transition co-ordinator and structured transition program, and giving the opportunity for patients to get to know the adult health care providers have all shown good results (23). Overall, when young adults are supported during the transition period, clinic attendance, and glycemic control can be maintained or improved, and diabetes-related complications reduced (23).

Whilst much can be learnt from established transition models, the advent of better personal communication technology affords the potential for new paradigms to enable transition. Results from a randomized clinical trial evaluating an 8-month technology based transition intervention MD2Me has had promising results in a mixture of patients with CF, T1D, and inflammatory bowel disease (24). The focus was on generic disease management skills rather than disease specific skills, with 81 patients recruited and randomized to receive standard care or MD2Me. The intervention arm consisted of a 2-month intensive web-based and text-delivered disease management and skill-based intervention followed by a 6-month review period. Participants also had access to an automated SMS algorithm that provided disease management decision support and was a communication portal with the health care team. MD2Me patients showed significant improvements in performance of disease management tasks, health-related self-efficacy, and patient-initiated communications compared with controls. Technology based interventions may be particularly relevant to patients on LTV, given that the majority of ventilators now have the capability to remotely transmit ventilator data to the clinical team and clinicians can also remotely adjust ventilatory settings.

## Unique Aspects of LTV Patients

As mentioned earlier, LTV patients tend to be patients with complex needs. A large proportion are patients with NMD who are often transitioning at a time when natural disease progression is resulting in loss of independence, an increasing reliance on LTV, and the emergence of a raft of new health problems. A survey of patients with Duchenne muscular dystrophy (DMD) and their families regarding transition found that approximately a third of the young men in the study were not in any kind of education, training, or work (25). The majority thought they were unlikely to get jobs and those who had tried, reported facing insurmountable difficulties relating to employer attitudes, access problems, and a lack of specialist advice regarding employment. A smooth transition is crucial for both their physical and psychological health. It is not just the mental health of patients at stake. Eighty percent of the parents of these young adults reported clinical levels of depression and anxiety. They felt that appointments in later childhood and early adulthood seemed to focus on charting the decline and deterioration of the health of the young men with DMD, something both parents and patients found to be a deflating and demotivating experience. However, issues such as ceilings of care and views regarding tracheostomy insertion do need to be explored and the balancing of these contrasting needs is difficult and requires both experience and adequate resources.

Another group of patients are those with severe cerebral palsy, who are on LTV for nocturnal hypoventilation. They may have severe neurocognitive impairment, seizures, and often require gastrostomy feeding. Given these needs, they are often heavily/completely reliant on their parents for their cares. This does not easily fit into the typical model of adult health care where there are expectations that it is the patient who directs and is responsible for their own care. Given the diversity of diseases that are now benefiting from LTV, the individual differences in disease progression between patients and the variations in both local and national care provision, transition for LTV patients cannot be a one size fits all approach, but needs ideally to be realized on an individual basis.

## Transition in LTV Patients—Current Situation

In 2011, the American Academy of Pediatrics (AAP) published a clinical report on supporting the health care transition (HCT) from adolescence to adulthood in the medical home (26). It proposed an algorithm containing action steps such as discussion of transition policy, initiation of a transition plan, and review of this plan at specific time points. It incorporated an assessment of transition readiness—this assessed the skills the adolescent would need to acquire in preparation for the change from pediatric parent supervised health care to patient centered adult health care with the legal assumption of self-determination at age 18 years. It also highlighted the importance of clarifying issues such as medical decision-making responsibilities and adult consent and confidentiality policies. On the back of this report, the “Six core elements of health care transition,” a structured process that can be customized for use in different settings and applied to different types of transition care models was developed. The six sequential core elements are:

1) Discussion of transition policy
2) Transition tracking and monitoring
3) Transition readiness and/or orientation to adult practice
4) Transition planning
5) Transfer of care
6) Transition completion and ongoing care with the adult healthcare team.

Similarly, the Canadian Thoracic Society have published clinical practice guidelines on pediatric home mechanical ventilation and the recommendations they made regarding transition are summarized in Table 1 (27).

**Table 1:** Recommendations for transition of patients on LTV.

1. Transition is an ongoing process and planning should start early in childhood. a. The adolescent and his/her caregivers should have a good understanding of his/her medical condition and what is required for transition into the adult healthcare system. If feasible, based on the developmental level of the child, the patient should be involved in planning discussions in a way that is meaningful to them. b. The adolescent should be involved in planning discussions and assume the management of their condition to the extent that they are able, prior to transfer of care. c. Providers should facilitate and encourage the attainment of self-esteem and self-confidence to allow successful transition and assumption of as much independence as their condition allows.
2. A formalized approach to transition is needed a. A formal transition plan should be developed in collaboration with the adolescent and his/her family. b. The transition program should be agreed upon and coordinated by both the pediatric and adult healthcare teams. c. Joint transition clinics with the pediatric and adult healthcare teams are recommended.
3. Should the adolescent be unable to provide informed consent, discussions regarding whom should have this responsibility need to be clarified. Conversations regarding goals and ceilings of care should be had with the pediatric team and the details of these conversations need to be clearly communicated to the adult healthcare providers.
4. Any differences in care in the adult setting from their usual pediatric care, need to be highlighted. The adolescent and his/her caregivers need to be made aware of any changes to homecare support.
5. A named key worker to oversee and co-ordinate transition support, help the young person navigate adult services, be the link between them, and the various practitioners involved in their support, advocating for them when necessary, can be invaluable.

*Adapted from Ian MacLusky and Krista Keilty on behalf of the CTS Pediatric Home Ventilation Guidelines Panel. Section 12: Transition from pediatric to adult care. Can J Res Crit Care Sleep Med. (2018) 2(Suppl 1):83–7. doi: 10.1080/24745332.2018.1494992*.

Despite this, when pediatric pulmonary program directors in the US were surveyed on the transition process of their respiratory technology dependent patients in 2015, 78.1% of respondents reported their work place did not use a standard protocol for transition, of which 41.4% had no process in place at all, only 13.8% assessed transition readiness and only 24% track the transition process until the first visit with the adult team (28). These results revealed just how much progress still needs to be made in this group of patients.

## Facilitators and Barriers to Transition for LTV Patients

Dale et al.'s (29) recent qualitative research explored the transition experience of patients who had undergone the home mechanical ventilation (HMV) transition program of a tertiary children's hospital to their partnering adult hospital. Identified factors that aided transition include early transition discussion, joint pediatric—adult HMV clinic visits, written information about adult services, and communication training for the adolescents to improve their capacity to give accurate medical histories and discussions of their needs with the clinical team. The barriers identified include lack of referral to other medical specialists, difficulty co-ordinating appointments across multiple adult specialists, and health care settings, inadequate information on adult community funding structures, and limited involvement of family doctors. The next section explores these barriers in more detail.

## Care Delivery and Co-Ordination

In some countries, one of the most significant barriers is the lack of adult health care teams to whom LTV patients can be transitioned to. Similar to the situation in CF a few decades ago, only a limited number of adult physicians have experience in looking after these patients and the multidisciplinary team set up which patients and their families are used to in pediatric care may only exist in a few tertiary centers. Some pediatric teams are therefore having to continue to look after these patients well into adulthood (30). Development of these adult services and training of the next generation of health care professionals needs to be a priority. From the CF experience, we know that it is possible to rise to this challenge—when the CF Foundation in the United States mandated that approved CF programs must provide adult CF services by the year 2000, it galvanized rapid progress and development of adult CF service provision to the high standards achieved now. It must be cautioned though, that the relative rarity of some diseases of patients requiring LTV makes the advocacy that CF relied on potentially less potent. Thus the potential unification of multiple small patient advocacy groups to address this must be considered.

In addition to being under the care of the pediatric pulmonologist who manages the respiratory and LTV aspects of his/her care, patients are often also under the care of several other specialties. For example, a neuromuscular patient will also typically be under the care of the neuromuscular pediatrician, spinal surgeons for scoliosis, orthopedic surgeons if they have dislocated hips, and may also see a cardiologist if their condition is associated with cardiomyopathy, gastroenterologist, or urologist. The transfer to the respective adult clinicians ideally should be done sequentially rather than all at the same time and thus requires careful advance planning. Feedback from our patients suggests that they appreciate having input into their sequence of transfer of care, often preferring transfer of services which were least engaged first, followed by the more closely involved services, often choosing to transition from respiratory LTV services last. Some adult centers are learning from the pediatric model and have started to develop “one stop” multidisciplinary services where patients are not only seen by the LTV team, but also in conjunction with neurology, orthopedics, gastroenterology, cardiology, and urology as required. This will help considerably with the problem of fragmentation of subspecialist input.

In certain health care systems, there may also be insurance coverage and cost of care barriers. For example in the US, pediatric hospitals may not always participate in the same medical insurance plans as their partnering adult hospitals. Patients would then have to face the choice of changing insurance providers, with the attendant changes to durable medical equipment, nursing, and pharmacy services, or stay with the same insurance company, but transition to another adult hospital covered by the insurance plan, which might not have the optimal pulmonary expertise or as close links with the pediatric team.

Transfer to adult care is best conducted when the youth's health condition is stable. Some adolescents on LTV are amongst the most vulnerable patients and may have periods with frequent or prolonged hospitalizations which can delay transition. It is therefore important to factor in unexpected, unavoidable delays, and not leave transition to the last minute. Pediatric wards and pediatric intensive care units often have age cut offs above which they will not accept a patient and should the young person become unwell and require an acute admission, being admitted to an adult ward without having completed the transition process is less than ideal.

## Patient Preparation and Engagement

Preparation for transition needs to occur early on—as adolescence approaches, developmentally appropriate education needs to be integrated into the patient's care plan, to better enable their understanding of their medical condition, and encourage more active participation and gradual assumption of responsibility for their medical care. Adolescence is a time when health is often not on the list of top priorities for a young person. When starting college and making new friends, remembering to change the filter on one's ventilator, and getting it serviced can easily be forgotten.

If available, having a designated adolescent ward or clinic can be a helpful stepping stone towards integration into adult services. Adolescent patients can feel they “don't fit in” if admitted to the same ward as a 70 year old on LTV for chronic obstructive pulmonary disease. Recent feedback from one family stated their teenage son was incredibly upset after attending an adult clinic as whilst waiting to be seen, they met an older patient with the same underlying condition, who had significantly more morbidity, being much weaker, and needing ventilation via a mouthpiece during the day as well as overnight. Woken to the potential ramifications of his condition, he struggled to come to terms with this, eventually needing referral for psychological support.

Over the years, these young people and their families have built up relationships with the nursing staff, clinicians, and health care professionals. It can emotionally be very difficult to break off contact and learn to trust a new set of clinicians. Young people and their careers have also become very familiar with the routines in clinic and on the ward and know how to access the various services available. Getting to know a new adult health care system can be challenging, and even simple logistical issues such as arranging transport to attend hospital appointments may not be straightforward when one is non-ambulant, given the attendant need for multiple equipment such as ventilators, cough assist, and suction machines, as well as feed pumps. Having a named key worker to oversee and co-ordinate transition support, help the young person navigate adult services, be the link between them, and the various practitioners involved in their support, advocating for them when necessary, can be invaluable.

Any differences in organization, investigations, and treatment between the pediatric and the adult center should be clearly documented and explained to the patient before transition. The adult team may use slightly different medications or therapies and some practices may vary. This needs to be clearly explained to families so they know what to expect, and applies to all aspects of the multidisciplinary care. Ceilings of care and healthcare expectations should be discussed during transition and revisited—the young person should be reassured that just because they have made a certain decision at this point in time, circumstances may change and they can change their mind in the future.

When handing over to the adult team, in addition to medical details such as ventilator settings, medications, and sleep study results, patients often appreciate it when the adult team are interested in getting to know them as a person as well. This is reflected in the transition paperwork from our center by a section titled “All about me,” where the young person writes down the things which they feel are important for the adult team to know about them. This has proved to be very helpful because it can stimulate topics of discussion when patients first meet the adult team, which can break the ice and help establish a good rapport.

## “Social” Aspects of Transition

While the clinical complexities of patients are often the predominant focus for medical personnel, these should not overshadow the “social” aspects of transition. These are slightly outside the remit of this review, but the experience of those undergoing transition in cerebral palsy (CP) is salutary in this regard. For in CP, the primary barriers to transition identified have been the navigation of the complex systems of services and supports, the lack of information and resources, and finally societal prejudices and stigmas toward individuals with disability (31–33). The parallel for LTV patients is obvious, with examples including the financial implications of the potential transfer of funding from the parents directly to the patient. Similarly, receiving educational institutions/employers often require clear guidance on the patient's functional capacity and degree of technology dependence to ensure that this can safely be accommodated. Sadly, employers or tertiary institutions are often initially reluctant to address these demands despite clear regulatory requirements. It has been our experience that patient support groups and charities can often be incredibly helpful in bridging the gaps in understanding seen in the wider non-medical context of further education/employment. Their role as a source of specific relevant information and capacity to facilitate communication often makes them invaluable partners for patients and medical teams during and after transition. For example, in the UK, Action Duchenne ran a Transition to Adulthood project providing information for patients regarding further education, training, and/or employment options, independent living including housing options, the impact of good health care provision including the importance of advice on sexuality, health, and relationships. Contact for families with disabled children provides parents with information such as on the UK Care Act 2014, explaining the important legal duties on local authorities about what must happen when a child makes the transition from children's to adult services and the rights of the young persons and careers. The majority of organizations also have a helpline for advice where workers already known to families can provide support with no predetermined age cut off. The key to the best utilization of these NGOs from a medical perspective is the realization of their existence, so suitable sign posting, if agreeable to the patient/family, can allow the development of a working relationship prior to transition. Parent/careers are also often reaching an age where they may themselves have health issues and may not be able to care for the patient in the same way as before and the familiarity of people whom they perceive have the long term interests of the family at heart can be of great benefit. Special mention should also be made regarding patients who are under the care of social services and/or those who lack the capacity for autonomous decision making, as the issue of power of attorney needs to be addressed before transition can be completed. The ultimate holy grail is a holistic approach to transition where preparation for transition meetings ideally should also include wider community agencies such as community health teams, education, and social services.

## Future Research

When the AAP transition guidelines were updated in 2018, new recommendations pertaining to research were added (34). These highlighted the importance of developing a stronger evidence base, examining transition outcomes in terms of population health, patient and family experience, healthcare use, and cost savings. Using a three stage Delphi process, members of the Health Care Transition Research Consortium identified the top 10 health care transition outcomes of adolescents with special healthcare needs (35). Quality of life was rated the most highly. The others included understanding the medical condition and its complications, knowledge of medications, self-management, adherence, understanding health insurance, attendance at medical appointments, having a medical home, avoidance of unnecessary hospitalization, and having a social network. Achieving consensus is an important first step toward high quality transition research and thus it is hoped that this ranking will help provide a framework for future research on transition programs for LTV patients.

## Conclusion

While transition services for LTV patients are, for the most part, often still in their infancy, there are some obvious themes already emerging. The most important of these is that done effectively, transition is a process, not a single event of transference of care. The insights gained from examination of other chronic diseases (summarized in Table 2) highlight the absolute requirements, where possible, for better education and communication. The later not only reflecting the conversations between medical professionals and patients, but also between the pediatric and adult teams involved in planning and coordinating this process. Strong advocacy from both patient groups and medical professionals is needed to stimulate the system wide changes still very evidently required. Key amongst these from a systemic medical perspective is the widespread establishment of multidisciplinary adult healthcare teams and the training of the next generation of health care professionals experienced in looking after patients on LTV. For although all patients, whether manifesting complex needs or not, require some degree of individualized planning, this can only happen if there is a systemic recognition of the need for greater collaborative care partnerships between pediatric and adult clinicians.

**Table 2:** What can we learn from the transition experiences of other medical conditions?

(1) No one transition model has been shown to be clearly superior to the rest. Adopting the model that best fits with how local healthcare is organized, whilst bearing in mind the recommendations in Table 1 seems a pragmatic option. (2) Formal training in transition should be part of the training of pediatric and adult physicians. (3) Investment of time and resources into patient education can improve understanding of their medical condition and facilitate development of self-efficacy. (4) Strong advocacy can result in rapid progress and development of high quality adult multidisciplinary teams and transition services. (5) Discussions about transition should be started early. (6) There needs to be close cooperation between the pediatric and adult centers, ideally with joint adoption of the same diagnostic and treatment protocols tailored to specific age groups, to ensure a transition that is as seamless as possible. (7) A formal transition clinic where there is participation of both pediatric and adult teams is important. (8) A comprehensive formalized transfer report including input from every member of the multidisciplinary team must be provided. (9) With adequate support during the transition period, clinic attendance and health can be maintained. A named key worker to oversee, coordinate, or deliver transition support, and be the link between the young person and the various practitioners involved can be very helpful. (10) Technology based interventions may be a helpful adjunct, especially since the majority of ventilators now have the capability to remotely transmit ventilator data to the clinical team and clinicians can also remotely adjust ventilatory settings.

## Author Contributions

All authors listed have made a substantial, direct and intellectual contribution to the work and approved it for publication.

## Conflict of Interest

The authors declare that the research was conducted in the absence of any commercial or financial relationships that could be construed as a potential conflict of interest.
